# Impact of Foods and Dietary Supplements Containing Hydroxycinnamic Acids on Cardiometabolic Biomarkers: A Systematic Review to Explore Inter-Individual Variability

**DOI:** 10.3390/nu11081805

**Published:** 2019-08-05

**Authors:** Daniela Martini, Laura Chiavaroli, Antonio González-Sarrías, Letizia Bresciani, Susana A. Palma-Duran, Margherita Dall’Asta, Georgia-Eirini Deligiannidou, Marika Massaro, Egeria Scoditti, Emilie Combet, Viktorija Maksimova, Mireia Urpi-Sarda, Christos A. Kontogiorgis, Cristina Andrés-Lacueva, Eileen R. Gibney, Daniele Del Rio, Christine Morand, Mar Garcia-Aloy, Ana Rodriguez-Mateos, Pedro Mena

**Affiliations:** 1 Human Nutrition Unit, Department of Veterinary Science, University of Parma, 43121 Parma, Italy; 2 Human Nutrition Unit, Department of Food & Drug, University of Parma, 43124 Parma, Italy; 3 Department of Nutritional Sciences, Faculty of Medicine, University of Toronto, Toronto, ON M5S 1A8, Canada; 4 Laboratory of Food and Health, Research Group on Quality, Safety and Bioactivity of Plant Foods, CEBAS-CSIC, 30100 Murcia, Spain; 5 School of Medicine, Dentistry and Nursing, College of Medical, Veterinary and Life Sciences, University of Glasgow, Glasgow G31 2ER, UK; 6 Laboratory of Hygiene and Environmental Protection, Department of Medicine, Democritus University of Thrace, 68100 Alexandroupolis, Greece; 7 National Research Council (CNR), Institute of Clinical Physiology (IFC), 73100 Lecce, Italy; 8 Department of Applied Pharmacy, Faculty of Medical Sciences, Goce Delcev University, 2000 Stip, Macedonia; 9 Biomarkers & Nutrimetabolomic Laboratory, Department of Nutrition, Food Sciences and Gastronomy, XaRTA, INSA, Faculty of Pharmacy and Food Sciences, University of Barcelona, 08921 Santa Coloma de Gramenet, Spain; 10 CIBER de Fragilidad y Envejecimiento Saludable (CIBERFES), Instituto de Salud Carlos III, 08028 Barcelona, Spain; 11 UCD Institute of Food and Health, University College Dublin, Dublin 4, Ireland; 12 School of Advanced Studies on Food and Nutrition, University of Parma, Parma, Italy and Microbiome Research Hub, University of Parma, 43124 Parma, Italy; 13 Université Clermont Auvergne, Institut National de la Recherche Agronomique (INRA), Unité de Nutrition Humaine (UNH), CRNH Auvergne, F-63000 Clermont-Ferrand, France; 14 Department of Nutritional Sciences, School of Life Course Sciences, Faculty of Life Science and Medicine, King’s College London, London SE1 9NH, UK

**Keywords:** polyphenols, caffeoylquinic acids, hydroxycinnamic acids, coffee, potato, artichoke, cereals, dietary supplements, interindividual variability, cardiometabolic health

## Abstract

Plant-based diets rich in bioactive compounds such as polyphenols have been shown to positively modulate the risk of cardiometabolic (CM) diseases. The inter-individual variability in the response to these bioactives may affect the findings. This systematic review aimed to summarize findings from existing randomized clinical trials (RCTs) evaluating the effect of hydroxycinnamic acids (HCAs) on markers of CM health in humans. Literature searches were performed in PubMed and the Web of Science. RCTs on acute and chronic supplementation of HCA-rich foods/extracts on CM biomarkers were included. Forty-four RCTs (21 acute and 23 chronic) met inclusion criteria. Comparisons were made between RCTs, including assessments based on population health status. Of the 44 RCTs, only seven performed analyses on a factor exploring inter-individual response to HCA consumption. Results demonstrated that health status is a potentially important effect modifier as RCTs with higher baseline cholesterol, blood pressure and glycaemia demonstrated greater overall effectiveness, which was also found in studies where specific subgroup analyses were performed. Thus, the effect of HCAs on CM risk factors may be greater in individuals at higher CM risk, although future studies in these populations are needed, including those on other potential determinants of inter-individual variability. PROSPERO, registration number CRD42016050790.

## 1. Introduction

Cardiometabolic (CM) diseases include cardiovascular diseases (CVD), diabetes and their related risk factors, such as obesity, insulin resistance, impaired glucose tolerance, dyslipidemia, hypertension and central adiposity. Recently, the Global Burden of Disease study stated that CM diseases are the leading causes of death worldwide, mainly triggered by the rise in biomarkers of metabolic syndrome (MetS), such as waist circumference (WC), fasting blood glucose, triglycerides (TG) and blood pressure (BP) [1]. A diet rich in plant-based foods, such as fruit and vegetables, might decrease the risk of CVD in all individuals [2,3,4]. Plant foods are generally rich in fiber, vitamins, minerals and other bioactive compounds like glucosinolates, carotenoids and phenolic compounds. Phenolics are a family of over 5000 compounds, which can be classified as flavonoids (i.e., flavonols, flavanones, flavones, flavan-3-ols, isoflavones and anthocyanins) and nonflavonoids (including phenolic acids, lignans, hydrolyzable tannins and stilbenes) [5,6]. Among nonflavonoids, phenolic acids are low molecular weight compounds composed of at least one organic carboxylic acid bound to a phenol ring. Phenolic acids can be in turn classified into hydroxybenzoic or hydroxycinnamic acids (HCAs), if deriving from benzoic or cinnamic acid, respectively. Regarding HCAs, *p*-coumaric, ferulic and caffeic acids are the most abundant in plant-based foods, but can also be bound to other organic acids, such as quinic acid, by forming coumaroylquinic, feruloylquinic or caffeoylquinic acids (CQAs), mainly known as chlorogenic acids (CGAs) [6,7,8].

HCAs are present in several food sources, including coffee, cereals, potatoes and artichokes and, for this reason, they are generally ingested daily in high amounts with an estimated intake of ~200 mg/day in adults [9,10]. Once ingested, only a small part of HCAs are absorbed in the upper gastrointestinal tract, while the majority reaches the colon unmodified, to be then subjected to extensive catabolism by the gut microbiota [11]. These compounds and their metabolites, such as dihydrocaffeic and dihydroferulic acids, have been associated with a wide range of biological activities, which may contribute to the prevention of CM diseases [12]. For instance, HCA consumption has been shown to exert anti-inflammatory activities, and play a role in glucose and lipid metabolism regulation in in vivo animal and human studies [13]. However, despite the extensive literature available on this topic, the evidence regarding the role of HCAs on CM health is not consistent [5,6,13,14]. This can be partially due to the highly heterogeneous characteristics of human study participants recruited in the different investigations [15].

An increasing number of studies investigating the health effects of plant food bioactives have indeed shown that the response to the consumption of phenolic compounds may vary among different individuals [16,17,18,19]. This inter-individual variability may be due to genetic and non-genetic factors, such as age, sex, ethnicity and gut microbiota, and it may involve variations in human metabolism and in the bioavailability of plant food bioactives [16,17]. These aspects may affect the biological response on CM health outcomes and, as a result, some individuals may benefit more than others from the health effects of these compounds. To better elucidate the impact of inter-individual variability on the effects of phenolics on human health, some research groups have reviewed the current literature related to specific classes of phenolic compounds, namely the class of flavonoids, including flavanols and flavonols, suggesting that these bioactives might be more effective in specific subgroups [18,19].

However, the inter-individual variability in the efficacy of the main dietary phenolic acids, namely HCAs, which are consumed in abundance in coffee and cereals, has not been fully explored. Therefore, the aim of this systematic review was to provide an overview of the randomized controlled trials (RCTs) evaluating the effect of HCAs on markers of CM health, in order to increase the knowledge about the impact of the inter-individual variability in the responsiveness to the consumption of this class of phenolic compounds.

## 2. Materials and Methods

### 2.1. Search Strategy and Study Selection

This systematic review was conducted following the Cochrane Handbook for Systematic Reviews of Interventions [20] and the Centre for Reviews and Dissemination’s guidance for reviews in health care [21] and was reported in line with the PRISMA (Preferred Reporting Items for Systematic Reviews and Meta-Analyses) statement guidelines (Supplementary Table S1) [22]. The review protocol was registered in the International Prospective Register of Systematic Reviews (PROSPERO, registration number CRD42016050790) [23].

The systematic literature search was conducted using PubMed (http://www.ncbi.nlm.nih.gov/pubmed) and the Web of Science (http://apps.webofknowledge.com) databases on December 2016 (updated December 2018), using the syntaxes reported in Supplementary Table S2. Electronic searches were supplemented with manual searches of references from included studies and reviews on similar topics. Studies were included in the present systematic review, based on the PICOS process (Supplementary Table S3), if (i) they were RCTs investigating the effect of the consumption of HCA-rich foods (artichoke, coffee, potato and cereal-based foods such as rye) or HCA extracts (i.e., containing HCAs extracted from one of the above-mentioned HCA-rich foods) in humans, where this was compared to a control, which was not high in HCAs; (ii) provided a quantitative characterization of the HCA content and (iii) reported on one or more of the following CM markers: Body mass index (BMI), WC, systolic and diastolic BP, TG, high-density lipoprotein cholesterol (HDL), low-density lipoprotein cholesterol (LDL), flow-mediated dilation (FMD), blood glucose, blood insulin, glycated hemoglobin (HbA1c), platelet aggregation or exercise capacity.

Exclusion criteria included (i) the presence of a co-intervention (e.g., physical activity) or other confounding factors; and (ii) not reported in a European language. No restrictions for the characteristics of study participants (e.g., age, sex and health condition) were applied.

### 2.2. Data Extraction

Two author-pairs independently assessed the studies for their inclusion. Disagreement between authors was resolved through consultation with a third author (PM) who performed an independent assessment to reach a consensus. Data were extracted from each identified study using a standardized form and the following information was collected: Name of first author; year of publication; study location; number of participants/controls; characteristics of intervention and placebo/control arms; characteristics of test food (e.g., HCA content); potential factors (e.g., sex, pathophysiological status) influencing the heterogeneity in the responses to the supplementation with HCA-rich food/extracts [24]; outcomes (BMI, WC, systolic and diastolic BP, TG, HDL, LDL, FMD, blood glucose, blood insulin and HbA1c); main findings. Studies were also assessed for whether there were any assessments made to explain inter-individual variability, including subgroup analyses (e.g., for age, pathophysiological status, sex, dietary patterns or specific polymorphisms). For these studies, the above-mentioned data were collected for each subgroup.

Data were then summarized by a qualitative assessment based on the results reported in individual studies. For each outcome assessed, studies were grouped by those significant or not and baseline levels of the outcome were used to compare studies. For studies that explored inter-individual variability in their own analyses, the results for each subgroup were assessed and summarized.

### 2.3. Risk of Bias

Risk of bias of the individual studies was assessed independently by two authors following the Cochrane Risk of Bias Tool [20]. The following categories were assessed: 1. Sequence generation and allocation sequence concealment (selection bias), 2. blinding of participants and personnel (performance bias), 3. blinding of outcome assessment (detection bias), 4. incomplete outcome data (attrition bias) and 5. selective outcome reporting (reporting bias). For each study, each category was assessed as either “Low risk of bias”, “High risk of bias” or “Unclear risk of bias”.

## 3. Results

### 3.1. Study Selection

**Figure 1:**
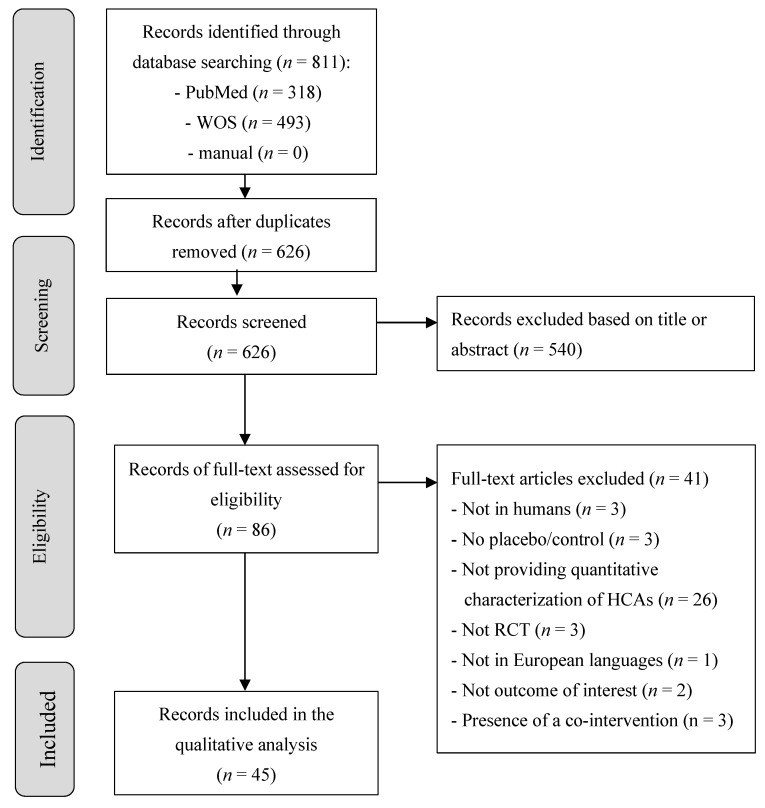
Flowchart of the study selection process. Legend: HCAs: Hydroxycinnamic acids; RCT: Randomized controlled trial and WOS: Web of Science.

The study selection process is shown in Figure 1. A total of 811 records were identified through the database search. After removing 185 duplicate articles, 626 studies were screened and 540 were excluded based on the title or abstract. A total of 86 eligible records went under the full text screening process, after which 41 records were excluded (Figure 1). Forty-five publications met eligibility criteria, providing data on a total of 44 unique RCTs, which were assessed in the qualitative analysis. The difference between the number of publications and the number of RCTs was due to the fact that three publications were on the same RCT but reported on different CM risk factors and, in one case, one publication reported results on two different RCTs.

### 3.2. Characteristics and Risk of Bias of the Included Studies

The main characteristics of the included studies are reported in Table 1 and Table 2. Out of the 45 included publications, 20 of them investigated the acute (i.e., single dose) effects of HCAs, which provided data on 21 RCTs (Mills et al. [25] included two different RCTs in the same publication). The remaining 25 publications investigated the chronic (2–16 weeks) effects of HCAs, which provided data on 23 RCTs (Martínez-López et al. 2018, Sarriá et al. 2016 and Sarriá et al. 2018 [26,27,28] provided data on different outcomes from the same RCT).

Most of the RCTs were performed in Japan (*n* = 11), followed by the United States of America and the United Kingdom (*n* = 5), Australia and Iran (*n* = 4), Italy (*n* = 3) and Germany, Denmark, Norway, Sweden, Finland, Greece, Thailand, Colombia, Mexico, Switzerland, Spain and Austria (*n* = 1).

For either acute or chronic studies, coffee was the most commonly investigated product, used as the HCA-source in 30 RCTs either as a beverage (*n* = 27), coffee polyphenols (*n* = 2) or as a green coffee extract (GCE; *n* = 1). Conversely, artichokes (*n* = 3), cereals (i.e., rye and wheat, *n* = 3) and potatoes (*n* = 2) were the least used sources of HCA. Pure compounds were also tested (*n* = 6), including both 3- and 5-CQA, ferulic acid or a mix of CGAs.

Risks of bias across the studies and within individual studies are shown in Supplementary Figure S1 and Supplementary Figure S2, respectively.

Studies often lacked details useful to make a judgment, mainly no mention of whether there was allocation concealment or blinding of outcome assessment, resulting in the majority of studies with domains predominantly assessed as unclear risk of bias. Conversely, there was very little high risk of bias observed where 0/21 acute studies and 4/23 chronic studies had only 1/5 domains (one study had two domains) rated as high.

### 3.3. Impact of Hydroxycinnamic Acids on Cardiometabolic Markers

#### 3.3.1. Acute Studies

Table 1 reports the main results obtained from the 21 acute studies evaluating the impact of HCA-rich foods or dietary supplements (pure extracts) on CM health outcomes. As mentioned above, coffee was the main HCA-rich source considered (*n* = 11), while only few studies were focused on phenolic-rich extracts from coffee (*n* = 4), as well as on rye (*n* = 2), potatoes (*n* = 1) and pure CGAs (*n* = 3). Total doses of CGAs ranged from ~3 mg [29] to 600 mg [30,31] for the phenolic-rich foods and from 400 [32] to 900 mg [33] for pure compounds. The number of study participants ranged from *n* = 9 [34] to *n* = 41 [35], while mean age ranged from 23 [25,29,36] to 59 years [33,37]. Overall, studies were conducted on healthy individuals, except for two studies focused on overweight individuals [38] and on individuals with borderline (systolic BP (SBP): 130–139 mmHg, or diastolic BP (DBP): 85–89 mmHg) or stage 1 hypertension (systolic BP: 140–159 mmHg, or diastolic BP: 90–99 mmHg) [39] and one study on individuals who had self-reported gastrointestinal symptoms after eating cereal, particularly rye [40].

Among the outcomes, markers of blood glucose metabolism (i.e., postprandial glucose and insulin) were most often considered (*n* = 16), followed by BP (*n* = 8) and markers of endothelial function (i.e., FMD, *n* = 8). There were no studies focused on platelet aggregation or exercise capacity. For acute glucose metabolism, 16 studies reported a measure of postprandial glucose, 13 of which calculated an incremental area under the curve (IAUC) [29,30,31,34,35,36,38,40,41,42,43,44,45], while three of them evaluated the effect at various postprandial time points [37,39,46]. Of the 13 reporting an IAUC, only two reported a significant reduction in IAUC for glucose after a HCA-rich intervention compared to a control [36,44], whereas one study demonstrated an increase [43]. One of these two studies [36], conducted on 12 healthy young adults, found a significantly lower 2 h IAUC for glucose after consumption of 25 g sucrose with coffee enriched with GCE and containing ~432 mg CGAs, compared to 25 g sucrose in water. However, there was no effect when sucrose was consumed with normal or decaffeinated instant coffee containing ~220 mg CGAs [36]. The second study [44] was conducted in 12 healthy young adults who consumed one of three potato chips (from red, purple or white potatoes) containing up to ~360 mg CGAs and providing 50 g of available carbohydrates and compared to the consumption of 50 g available carbohydrates from plain salted wheat crackers. The 2 h IAUC for glucose was significantly lower after each of the potato chips compared to the cracker control. Nevertheless, it is possible that the observed differences here may be confounded by the greater fat or fiber content of the potato chips tested compared to the crackers. The one study that reported a significant increase in 2 h IAUC for glucose was that by Robertson et al. [43], in which they only found the increasing effect on IAUC glucose when caffeine was added to the test decaffeinated coffees in overweight men, using water as the control drink. No effect on IAUC glucose was found between the test drinks and control when the same amount of caffeine was added to each beverage. The study by Schubert et al. in 12 healthy adults, however, did not find an effect of decaffeinated coffee vs. water in either case when consumed without caffeine or when caffeine capsules were consumed [45]. However, Schubert et al. [45] used a standard breakfast with the test beverages, which included pancakes, butter and jam as a means to provide 48 g of carbohydrate as opposed to glucose load. Thus, the effects of caffeine on the glycemic response may differ when consumed with carbohydrates, which may affect its metabolism [47].

Of the three studies that only reported differences in postprandial glucose at various time points, one demonstrated a reduction at the peak glucose (30 min) after the ingestion of a coffee polyphenol extract providing 355 mg CQAs consumed with a meal compared to when the meal was consumed without the polyphenol extract in healthy men [46]. The other two studies found no differences between test and control groups in postprandial glucose response. However, one of these [39] in 19 middle-aged adults with borderline hypertension found a significant increase in postprandial glucose at 1 h and 2 h after the consumption of either the roasted coffee with CGAs or the coffee without CGAs. This study differs from the other two in that the test and control beverages were consumed alone, without a glucose load or meal as it was used in the other two studies, which may limit comparability as well as applicability.

Twelve studies reported a measure of acute postprandial insulin, ten of which calculated an IAUC [29,30,31,34,35,38,40,41,42,43] and two assessed the effect only at various postprandial time points [39,46]. Of the ten, one study reported a reduction in 3 h IAUC and one study an increase, while the others found no significant effects. The one study that found the reduction in IAUC was conducted by Rosen et al., which assessed five different varieties of rye made into breads and compared to a white wheat bread in 14 healthy adults [29]. Interestingly, there were only two rye bread varieties, which significantly reduced 3 h IAUC insulin compared to the white wheat control bread. These two breads differed from the other three rye breads in that one (Rekrut) had the highest soluble fiber content and the other (Amilo) had the highest insoluble fiber content, as well as higher levels of certain bioactives including caffeic and sinapic acid. Dietary fiber content was related to the early (0–60 min) lower levels of glucose and insulin and it has been previously demonstrated that breads made with endosperm rye, which are rich in soluble fibers and bioactives, reduce glycaemia whereas wheat breads enriched in rye do not [29]. Therefore, the bioprocessing may influence the metabolic effects of rye breads, as has been previously reported [48]. On the other hand, the study that reported an increase in IAUC for insulin by Rakvagg et al. [42], in 11 healthy young adults, only found a significant effect of the dark roast coffee (66.6 mg CQA) vs. control (water), but not of the light roast coffee (~400 mg CQA), so the increasing effect of the dark roast coffee may not be due to the CQA content but to some other confounding factors. This study was also the only one to administer the interventions 30 min before the glucose load, which may affect the interpretation and comparison of results. Of the two studies, which only reported the effect of HCAs on insulin at various postprandial time points, no significant effects were found [39,46].

Nine acute studies reported effects of HCA on BP [25,30,31,32,33,39,49], however only one demonstrated a significant reduction and one a significant increase while the remaining seven studies did not find significant effects. The study that demonstrated a reduction was performed in 23 healthy adults (mean age 52 y) [32], found a significant reduction in SBP and DBP over 3 h after consumption of 400 mg CGAs in 200 mL low nitrate water compared to plain water. It was not clear whether the control water was matched for nitrate content and thus, since nitric oxide (which nitrate is a precursor to) is a vasodilator, it may confound the effect on BP. The one study that found an increase was the one performed by Ioakeimidis et al. [49] in 24 healthy adults (mean age 33 y), who showed a significant increase in DBP and SBP over 2.5 h after consumption of a decaffeinated or caffeinated coffee (both with 81 mg of 3-CQA and 156 mg of 5-CQA) or caffeine tablets compared to a control of hot water. Interestingly, six of these nine studies assessing BP tested the effects of HCA when consumed alone (without a meal or glucose load) [25,32,33,39,49]. Only one study was done in a population with borderline or stage 1 hypertension (mean baseline SBP, 128 mmHg) [39], with the remaining in healthy adults (all with mean baseline SBP < 125 mmHg, except one study with 130.5 mmHg). Therefore, the applicability and generalizability of these results are limited.

Eight studies reported effects of HCAs on FMD with 50% (four studies) demonstrating a significant increase in FMD compared to control [25,30,46], and two studies demonstrating a significant increase in FMD within the HCA treatment [33,37], although not statistically significant compared to the control. Interestingly, the two studies that did not demonstrate a significant effect only assessed FMD at 2 h post-ingestion [32,39], whereas five of the six studies that found some significant effect assessed FMD between 4–6 h post-ingestion, which may be of importance since most of the HCAs are not metabolized until in the large intestine [5,6,11]. All studies were conducted in those who were healthy, other than one in borderline or stage 1 hypertension [39], with mean BMI in the normal range (21.8–25.6 kg/m^2^) and an average age of 45 y. Of the six studies that found some significant effects, four were conducted solely in men, with the other two having 67% and 38% men, in contrast to the two studies without significant effects, which had 58% and 17% men. Thus, these results may be indicative of a possible effect in men, although more studies assessing this are needed, specifically including women, with longer follow up for assessments (>2 h), and in higher risk populations.

In general, the acute studies demonstrated no effect of HCAs on glycaemia or BP, with some potential effects on FMD. Limitations of the comparability of these studies include the wide variability in terms of study design and methods of outcome assessment. Due to the vast majority of studies being conducted in healthy participants and with HCA coming mainly from coffee consumed alone, generalizability and applicability is also limited. From these studies it is unknown what the effects may be in higher risk populations, including those with impaired glucose tolerance or diabetes, and when HCAs are consumed as part of meals, which may be more common in real-world consumption.

#### 3.3.2. Chronic Studies

The main findings associated with the 23 chronic studies investigating the effects of HCA-rich foods on cardiometabolic markers are reported in Table 2. Similar to the acute studies, coffee was the most commonly investigated HCA-rich food (*n* = 15), followed by artichokes (*n* = 3), wheat (*n* = 1) and potatoes (*n* = 1), while extracts or pure compounds were the subjects of three studies. The HCA dose was sometimes difficult to identify (e.g., in some cases expressed as mg/kg body weight) but it generally ranged from 25 mg CGAs [50] up to 1200 mg CGAs/day [51]. The number of participants ranged from 10 [52] to 183 [53] while mean age ranged from 23 [52] to 54 years [54]. Most study participants were generally healthy (*n* = 11), and sometimes with pathophysiological conditions such as mild hypertension or hypertension (*n* = 4), non-alcoholic fatty liver disease (NAFLD, *n* = 1), MetS (*n* = 2), impaired glucose tolerance (IGT, *n* = 1), impaired fasting glucose (IFG, *n* = 1), hyperlipidemia or hypercholesterolemia (*n* = 3). Three studies recruited solely individuals with overweight or obesity. The duration of the studies ranged from two weeks [52] to four months [55], although most studies (*n* = 17) were 4–8 weeks long.

Lipid profile components (*n* = 22), blood glucose (*n* = 14) and BP (*n* = 13) were the CM outcomes most often considered. There were no studies assessing platelet aggregation or exercise capacity. As already observed in acute studies, results from the different investigations were often contrasting. Regarding blood lipids, 55% of the studies (12/22 studies) demonstrated a significant effect on at least one measure of the lipid profile (i.e., decrease in TG, LDL-C and total-C and/or increase in HDL-C) following HCA consumption [28,50,51,53,54,56,57,58,59,60,61,62]. Fifty percent of studies (9/18 studies) that measured both LDL and TC demonstrated a significant reduction in both [28,50,51,54,58,59,60,61,62], aside from one which found significance only for TC [60]. In comparing those nine studies reporting a significant reduction to those that did not, those that did had a greater baseline LDL level (~3.34 mmol/L vs. 2.91 mmol/L). Furthermore, those studies that did find a significant reduction were also slightly more overweight (BMI ~28.9 kg/m^2^ vs. 26.4 kg/m^2^). The average of the mean ages in each study was similar between these groups (~43 y) and the ratio of men to women in each study was ~37% in those studies that found an effect compared to 49% in those that did not. However, only one study [54] assessed the effect separately for men and women and found consistent effects between sexes, except for a reduction in TG, which was only found for women.

Considering the source of the HCAs consumed, in the nine studies that found a significant reduction in TC or LDL, seven used capsules of extracts rich in HCAs [51,54,58,59,60,61,62], one used instant soup enriched in GCE [50] and one used 6 g/d of a soluble green/roasted coffee blend [28]. This contrasts the nine studies that did not find a significant improvement on TC or LDL, in which six studies used brewed or canned coffee [53,56,57,63,64,65], one used a beverage enriched in GCE [30], one fruit juice enriched in GCE [66] and one GCE in capsules [67]. Interestingly, one of the studies that used brewed coffee found an increase in TC, as well as an increase in HDL, after the consumption of 750 mL/d of moderate roasted coffee in 84 healthy subjects for 12 weeks [56]. The authors suggested that this increase in TC may be the result of the diterpenes (i.e., cafestol and kahweol) in coffee [68], which are present in unfiltered coffee. The food source of HCAs (interfering effects of other compounds in unfiltered coffee) may have influenced the difference in results between these two groups of studies.

For markers related to glucose metabolism, ~30% of the studies demonstrated a significant effect on fasting glucose (6/19 studies) [26,51,60,62,67,69] or HbA1c (2/7 studies) [54,62] following HCA consumption. In comparing those six and two studies that found a significant reduction to those that did not, those that did had a greater baseline glucose level (~6.00 mmol/L vs. 4.91 mmol/L and 6.55% vs. 5.9%, respectively). Furthermore, those studies that did find a significant reduction were also performed on slightly more overweight individuals (BMI ~27.9 kg/m^2^ vs. 26.2 kg/m^2^). The average of the mean ages in each study was similar between these groups (~41 y) and the ratio of men to women at ~45%. Of the nine studies that reported results for insulin, none demonstrated a significant reduction, with one that found a significant increase [56]. For homeostatic metabolic assessment-insulin resistance (HOMA-IR), 57% (4/7 studies) found a significant reduction [54,60,62,67] and one study found a significant increase [56]. In comparing the four studies that found a significant reduction in HOMA-IR to the two that found no effect, the mean baseline HOMA-IR level was higher (4.03 vs. 2.19), with similar BMI (31.3 vs. 29.5 kg/m^2^), age (45 vs. 44 y) and ratio of men to women (35% vs. 41%). Furthermore, those four studies all used HCAs consumed in the form of capsules (ranging from 372 mg to 600 mg/d CGAs from GCE or artichoke) [54,60,62,67] whereas the other three studies all assessed the effect of HCAs consumed in brewed coffee (ranging from 9 mg/d CGAs to 216 mg/d CGAs). Interestingly, the one study of 84 healthy adults that found a significant increase in insulin and HOMA-IR [56], had a much lower difference in CGA between the two coffees (difference of 9 mg/d of CGAs) compared to the rest of the studies, and the authors noted that the concentration of CGAs upon analyses demonstrate that they did not differ between the two coffees [70], thus the differences in outcomes may be the result of other differences between the coffees (e.g., *N*-methylpyridinium (NMP)). Additionally, one of the two studies that did not find an effect of HOMA-IR [57], conducted in 116 overweight adults, also described a similar intervention of brewed coffees to compare which differed in CGAs by 9 mg/d, thus may also not have had an adequate difference in CGAs in order to assess effectiveness.

The beneficial effects predominating in higher risk groups are further supported if we look at those studies that were done in higher risk groups. For instance, the consumption of HCA-rich foods provided beneficial effects to study participants with impaired glucose metabolism, who are at a higher risk of type 2 diabetes mellitus. In fact, the oral consumption of 1200 mg CGA by 30 study participants with IGT for 12 weeks [51], or 600 mg artichoke extract by 55 participants with IFG for eight weeks [62], significantly reduced fasting glucose (from 5.7 ± 0.4 to 5.5 ± 0.4 mmol/L) as well as other parameters such as the insulinogenic index and the homeostatic metabolic assessment (HOMA) index (−11.7%), compared to controls. Similarly, a positive effect on fasting blood glucose was observed in two other investigations on individuals with NAFLD supplemented with 1 g GCE/day (500 mg CGA) for eight weeks [60], as well as in individuals with MetS who consumed two decaffeinated GCE capsules (372 mg CGA/day) for eight weeks [67]. Interestingly, contrasting results were observed in four trials with healthy study participants [26,69,71,72]. A statistically significant amelioration of fasting blood glucose (from 107.6 ± 3.0 to 99.0 ± 2.5 mg/dL) was evidenced when healthy individuals consumed 185 mL of a test beverage with 329 mg CGA for four weeks [69] or green-roasted coffee blends providing 510 mg HCA/day for eight weeks [26]. On the contrary, Vitaglione et al. [71] failed to find any effect on blood glucose following an eight-week consumption of wholegrain biscuits (70 g, ~130 mg HCAs as sum of ferulic, sinapic and coumaric acids), similarly to Robertson and colleagues [72], who found no differences after the consumption of four cups/day of instant coffee (44 mg CGAs/serving) for 12 weeks, compared to non-coffee consumers.

Regarding BP, most studies (62%, 8/13 studies) demonstrated a significant effect on either SBP or DBP resulting from the consumption of HCA-rich foods [28,50,53,64,65,66,67,73]. In comparing those eight studies that found a significant reduction to those that did not, those that did had a greater baseline SBP level (~138 mmHg vs. 118 mmHg). Those studies that did find a significant reduction were performed on slightly less overweight subjects (BMI ~26.3 kg/m^2^ vs. 27.8 kg/m^2^) with similar mean ages (~45 y) and ratio of men to women (~46 vs. 43%). No study completed subgroup analyses by BP status or by sex.

With regard to measures of adiposity, 29% (5/17 studies) demonstrated a significant reduction in BMI, body weight or waist circumference [51,53,61,62,67]. In comparing those studies that found a significant reduction to those that did not, those that did had a greater baseline BMI (29.8 kg/m^2^ vs. 27.0 kg/m^2^) and greater baseline waist circumference (106.2 cm vs. 97.7 cm). Those studies that did find a significant reduction had similar mean ages (~44 vs. 43 y, respectively) and lower ratio of men to women (~30% vs. 54%). Interestingly the populations of those studies that found a reduction included (*n* = 1) mild hypertension, (*n* = 1) obese, (*n* = 2) impaired glucose tolerance and (*n* = 1) metabolic syndrome.

Overall, the results from each of the outcomes assessed in the chronic studies consistently demonstrate there is a significant effect in those studies with participants with higher baseline levels of each risk factor for CVD. Therefore, HCAs may be more effective in those at higher CVD risk. 

#### 3.3.3. Inter-Individual Variability

Only seven out of the 45 studies took into account one or more determinants that might explain inter-individual variability, such as pathophysiological status (*n* = 4), sex (*n* = 2), dietary patterns (*n* = 1) or specific polymorphisms (*n* = 1). The main findings of these studies are reported in Table 3. It is worth noting that the role of polymorphisms was also considered by the study of Robertson and colleagues, but the considered polymorphism (on the CYP1A2 gene) was related to the caffeine metabolism and no insights in the metabolism or effect of HCAs were provided [72].

Among the studies investigating the putative role of pathophysiological status in the effects of HCA-rich sources, two acute studies stratified results based on the glycemic response [35] or the insulinogenic index (an index of pancreatic β-cell function) [46]. In the former, the consumption of 300 mg of decaffeinated green coffee beans (EDGCB) extract significantly lowered peak glycemic levels, but not plasma glucose IAUC, after ingestion of a 200 g carbohydrate rich-meal, compared to water [35]. Interestingly, when results were analyzed on a sub-set of 18 study participants categorized as having a high-glycemic response (i.e., those with the highest mean postprandial glucose level 30 min after consumption of the loading diet/placebo food), plasma glucose 2 h IAUC was reduced following the 100 mg EDGCB beverage compared to control. Similarly, plasma glucose after 30 min was significantly lower after the ingestion of both low (100 mg) and high (300 mg) intake of EDGCB beverages, compared to controls. Jokura and colleagues [46] focused on the insulinogenic index and its potential role as a determinant of the effect of HCA on postprandial hyperglycemia and vascular endothelial function. In all study participants, the ingestion of a meal with a coffee phenolic extract (355 mg CQAs) beverage significantly lowered the plasma glucose levels (*p* < 0.05) after 30 min and FMD response after 60 min compared to the same meal without the coffee polyphenol extract. However, when study participants were stratified by insulinogenic index, those with an index <0.88 had significantly lower postprandial blood glucose concentration at 30 min (*p* < 0.01) compared to control, whereas there was no effect in those with an index ≥0.88. Thus in those with lower β-cell function, and thus at greater risk of chronic disease such as diabetes, there was an effect of HCA. With regards to the assessment of FMD, the response seemed not to be influenced by the insulinogenic index, being significantly higher at 120 min in both strata compared to control.

The role of background lipid status in determining the effect of HCA-rich foods on CM markers was considered in one chronic study (two publications [26,28]) in which half of the recruited study participants had hypercholesterolemia. In this study, daily consumption of green/roasted coffee blend (344 mg CQAs and ~510 mg HCAs total, plus ~120 mg caffeine) for eight weeks resulted in decreased the levels of several CVD risk factors including SBP, DBP, blood glucose, HOMA-IR, total-C, LDL-C and TG. However, when the results were separated by hyperlipidemia status, SBP, DBP, total-C, LDL-C and TG were significantly reduced after coffee consumption only in those with hyperlipidemia. Thus, demonstrating potential effectiveness of CQAs in those subjects at high CVD risk.

Regarding sex differences, the eight-week supplementation of a CQA-rich artichoke leaf extract significantly increased HDL-C in primary mild hypercholesterolemic study participants (0.207 mmol/L) [54]. However, when sex groups were compared, an increase in HDL was only significant for males and reductions in TG were only seen for women. However, when groups were further stratified by median HDL level, there was a significant increase in HDL in women who had ≤median HDL. The impact of sex was also considered in a study enrolling 80 patients with MetS and screened for polymorphisms of cholesteryl ester transfer protein (CETP), which is directly related to MetS risk [58]. Although there was no interaction in the whole population between CETP gene mutation and response to 12-week ALE supplementation (~22 mg CQAs), the subgroup analysis revealed that only men with CETP gene mutation had significantly lower LDL-C levels after 12-week supplementation, compared to placebo.

The inter-individual variability associated with the dietary pattern was taken into account by Ioakeimidis and colleagues [49] in an acute study focusing on habitual coffee consumption. Results revealed positive effects of both caffeinated and decaffeinated coffee consumption (79 mg CQAs each) on markers of arterial stiffness in non-habitual compared to habitual coffee consumers (maximal differences of changes in responses by 4.5%). As some markers, not subject of this systematic review (i.e., pulse wave velocity and augmentation index), increased only after decaffeinated coffee consumption in non-habitual consumers, authors hypothesized that the effect was related to compounds other than caffeine and supported the potential role of coffee habituation as determinant of its effect on vascular function.

## 4. Discussion

There is a clear interest in the exploitation of phenolic-rich foods as potential modulators of markers of CM health, and the present review aimed at summarizing the main findings from RCTs focused on HCA-rich foods. The observed intervention effects had clinical relevance mostly in study participants at high CVD risk. Conversely, many studies performed on self-reported healthy individuals failed to find any effect of HCAs, increasing the heterogeneity of the results and making it difficult to draw any clear conclusion.

In the present review, only seven studies out of the 45 included publications considered one or more determinants of inter-individual variability in response to HCAs, while the remaining studies did not stratify results based on these parameters. This hinders our understanding of the role played by these determinants in the individual response to HCA-rich foods.

Despite limited, results from these seven studies suggest that some individual characteristics may influence the beneficial effect of HCAs. Firstly, determinants of health or pathophysiological status, like baseline cholesterol levels [26,28], insulinogenic index [46] or glycemic response [35] could play an important role in the variation among study participants in the biological response to HCAs regarding CM outcomes, being associated with an increased beneficial effect following HCA intake. This was not only demonstrated in the stratified analyses conducted by Martinez-Lopez et al., Sarriá et al., Iwai et al. and Jokura et al., [26,28,35,46] but also in the assessments conducted within this systematic review for all outcomes in the chronic studies, which demonstrated that effectiveness of HCAs, regardless of specific source, was greater in those at higher risk (i.e., greater baseline cholesterol, glycemic or SBP). This supports previous studies that suggested that the pathophysiological status can lead to inter-individual variation in response to polyphenols [74,75]. Some recent systematic reviews and meta-analyses have also indicated that the health status or BMI may influence the impact of several polyphenols (flavonols, flavan-3-ols, anthocyanins and ellagitannins) on blood lipid levels [18,19,24].

Regarding sex differences, the response to HCAs has been shown to be different between men and women following artichoke leaf extract consumption [54]. So far, a sex effect in response to plant-food bioactive compounds such as HCAs has been reported in very few studies [76], mainly focusing on flavanol-rich products, with some but limited differences in the response between men and women. However, results are often contrasting; for instance, a decreased augmentation index was observed only in women after cocoa consumption for four weeks [77], while the antioxidant status was improved mostly in men after a four-week consumption of ready-to-eat meals supplemented with cocoa extract [78]. Again, TC and LDL were significantly reduced only in females after intervention with flavanol-containing products [19]. Similarly to what happens for other individual characteristics, the lack of differences in the response among men and women after consumption of HCAs could be due to the lack of statistical power resulting from smaller sample sizes after the stratification process.

Genetic polymorphisms have also been shown to impact the effect of plant compounds from different sources [79], since they are often present in genes encoding for enzymes involved in the metabolism of these bioactives. Regarding coffee, mostly polymorphisms related to caffeine metabolism have been investigated, above all the cytochrome P450 as CYP1A2 accounts for about 95% of caffeine metabolism, with a high inter-individual variability in activity [80]. In the present review, only one study investigating variation based on polymorphisms (in CETP Taq 1B) was included [58], suggesting that genetic polymorphism may predict the lipid responsiveness to HCA intake.

An additional determinant, not explored in any of the studies included in the present review, may likely be age, which is the strongest independent cardiovascular risk factor. Overall, the impact of age on the response to the consumption of plant food bioactives has received very limited attention [17]. Differences due to age might depend on variability in absorption, distribution, metabolism and excretion (ADME), as recently showed by Alkhaldy and colleagues, who found variation in the urinary phenolic acid profile between younger and older adults after a polyphenol-rich meal [81].

The inter-individual variability in the biological response to the consumption of phenolic compounds, such as HCAs, may be partially related to the inter-individual variation in the bioavailability and metabolism of these compounds [16]. Several human studies have demonstrated that the plasma concentrations and the urinary excretion of phenolics or their derived metabolites can differ markedly between individuals following a similar intake of plant food bioactives, due to variances in their ADME [82,83]. Despite a small amount of some (poly)phenols being absorbed in the upper gastrointestinal tract (and being subjected to glucuronidation, sulfation or methylation by the gut epithelium and/or liver) most compounds reach the lower gastrointestinal tract unmodified, where they undergo extensive metabolism [83]. Thus, a key role is played by the gut microbiota, which may modify the structure of polyphenols, releasing these colonic catabolites into the portal vein towards the liver, where they can: (i) Directly enter the bloodstream, (ii) undergo phase I and II metabolism and then enter the bloodstream and (iii) undergo enterohepatic recirculation until complete detoxification [84].

This long and complex physiological mechanism and the influence of gut microbiota composition form the pathway through which different types and levels of metabolites enter circulation where they may have metabolic effects. This is supported by the work by Stalmach and colleagues who assessed the metabolic fate of CGAs from coffee [85] and demonstrated that ~1 h after the consumption of instant coffee, low nanomolar concentrations of CQA lactones and caffeic acid sulfates reached the plasma. Although it has been hypothesized that quinic-HCA linkage is hydrolyzed with consequently few HCA conjugates being absorbed [83], ~5 h after coffee consumption, gut-derived phase II metabolites were detected in much higher concentrations in plasma showing high inter-individual differences. Gut microbial esterases can rapidly hydrolyze the quinic-phenolic linkage and convert the HCA into dihydroxy forms and further metabolism of these compounds by gut microbial strains leads to the formation of smaller catabolites [83,86,87]. Thus, future studies assessing the profile of phenolic metabolites in circulation in addition to variations in gut microbiota composition would be highly valuable in understanding the potential effectiveness of HCAs. Among the 44 included RCTs, only the one by Mills and colleagues [25] considered circulating metabolites and found that FMD responses to coffee intake were closely paralleled by the appearance of CGA metabolites in plasma.

In the framework of an intervention study, these aspects are further complicated by considering that the same colonic metabolites can be produced after the consumption of different classes of phenolic-rich foods. For example, in vitro and in vivo studies have shown that after berry, coffee, cocoa or orange consumption, the phenolic metabolic degradation pathways reach some common intermediates, most of them HCA or hydroxybenzoic acid derivatives, which can be further metabolized into smaller compounds [83,88,89]. To avoid this further confounding factor, some researchers have applied different strategies, such as: (i) Asking participants to maintain their usual diet; (ii) telling participant to refrain from consuming selected foods, rich in (poly) phenol compounds similar to those of the intervention and (iii) advising a low-/free-polyphenol diet in the day(s) prior to sample collection.

The limitations of the present systematic review include that there were relatively few studies (*n* = 7), which explored potential determinants of inter-individual variability, thus there may be other variables that may influence the effectiveness of HCAs on CM biomarkers. Additionally, of those included studies, there was a wide range of methods by which HCAs were delivered, as well as ranges of doses, including those coming from coffee (plain coffee and green coffee extracts consumed in a variety of forms (soups, capsules, in a test beverage and in fruit juice)) and foods (purple potatoes, whole grain biscuits and artichoke leaf extract), which may also influence the effect of HCAs. Furthermore, some studies controlled for caffeine whereas others did not, and considering the majority of studies focused on coffee, this also potentially confounds the ability to determine the effect of HCAs. In addition, further efforts should be paid on the contribution of other phytochemicals to the observed effects when dealing with food products rich in different bioactive compounds. A good example may be coffee, where the presence and amount of some compounds like caffeine, trigonelline and HHQ may affect the response to the intervention [8,38,64]. Lastly, it may be a limitation that only two databases were used for conducting the search and thus may not have captured all existing studies on HCAs, however manual searches of the reference lists of all included studies, supplemented the search to minimize this potential issue.

Overall, the studies were characterized by a high variability in study design and methods of outcome assessment and, consequently, comparisons among findings of the different studies challenging. In addition to the health status of the volunteers, major sources of variability include the type of HCA-based product (e.g., pure compounds, foods and beverages) and the dose of HCAs, which broadly ranged in both acute and chronic studies. It is worth noting that, even when the same HCA-food source was used as test food (e.g., coffee) in different investigations, a high variability in terms of phenolic content was observed. This further supports the importance of an in-depth characterization of the phenolic profile of the test items and, when possible, taking into account the actual phenolic amount provided by a single serving [90,91]. In addition to the above-mentioned sources of variability, studies often lacked in information on determinants that are known to influence CM biomarkers, such as BMI, age, sex, smoking habits, dietary patterns, physical activity levels or specific polymorphisms. Improvements in reporting possible determinants of inter-individual variability are needed to make secondary data analysis feasible [92].

## 5. Conclusions

The present review summarized the main findings of RCTs investigating the effect of HCA-rich foods on CM health, with a particular focus on the determinants of inter-individual variability such as pathophysiological status, sex or specific polymorphisms. Generally, there are limited studies that conduct stratified analyses to explore factors leading to inter-individual variation in the effect of HCAs to modulate CM outcomes, thus limiting the ability to conclude which factors may modulate the effectiveness of HCAs. However, the present systematic review demonstrates that overall, HCAs have greater effectiveness, regardless of the variation in the source of HCAs, in those subjects with greater baseline levels of cardiovascular risk factors such as cholesterol, BP and glycaemia. Further high quality, well powered RCTs with a low risk of bias should be performed, reporting all characteristics that may influence the individual response to these compounds, including but not limited to sex, age, BMI, pathophysiological status, presence of genetic polymorphisms, smoking habits, physical activity level and dietary patterns.

## Figures and Tables

**Table 1:** Characteristics of the included acute intervention studies investigating the effect of HCA-rich foods on cardiometabolic markers.

Reference	Study Participants	Study Design	Intervention	Dose of HCAs	Control/Placebo	Main Findings
Beam et al. 2015 [41]	*n* = 10 male cyclists; age 26 ± 5 y; BMI 24 ± 4.3 kg/m^2^	CO	75 g of dextrose with 10 mg/kg bw of GC bean extract post-exercise	5 mg/kg bw CGA (325–450 mg CGA)	75 g of dextrose with 5 mg/kg bw caffeine	= blood glucose and insulin 2 h AUC, Matsuda Index
Boon et al. 2017 [37]	*n* = 12 (7M/5F); age 59.4 ± 6.4 y; BMI 24.7 ± 3.3 kg/m^2^	CO	(1) 18 g of DC in 200 mL of hot water taken after a 12-h fast; then again 2h later plus 75 g glucose drink (2) Note: Another treatment included 18 g of ground caffeinated coffee in 200 mL of hot water, taken after a 12-h fast; then again 2 h later plus 75 g glucose drink	(1) 287 mg CGAs (DC) (2) 300 mg CGA (caffeinated coffee)	200 mL of hot water, taken after a 12-h fast; then again 2 h later plus 75 g glucose drink	= 1 h FMD, 2 h glucose (at any time point) and 1 h or 2 h BP (based on a single reading) ↑FMD after caffeinated coffee vs. DC or control
Ioakeimidis et al. 2018 [49]	*n* = 24 (16M/8F); age 32.7 ± 9.3 y; BMI 26.4 ± 2.5 kg/m^2^ (habitual consumers) and 26.4 ± 2.5 kg/m^2^ (non-habitual consumers)	CO	(1) triple coffee espresso (75 mL) (2) triple DC espresso (75 mL)	81 mg of 3-CQA and 156 mg of 5-CQA	(1) 240 mg caffeine tablet (2) placebo (hot water, 75 mL)	↑peripheral SBP, DBP after DC, caffeinated coffee and caffeine tablet compared to placebo after 2.5 h
Iwai et al. 2013 [35]	*n* = 41 (22M/19F); age 34.8 ± 8.0 y; BMI 22.0 ± 3.1 kg/m^2^	CO	2 pieces of Onigiri (200 g carbohydrate) together with: (1) 100 mg or (2) 300 mg decaffeinated green coffee beans extract in 200 mL water	39 and 118 g/day (39.2 g CGA/100 g)	2 pieces of Onigiri (200 g carbohydrate) together with 200 mL water	↓glucose after 300 mg decaffeinated green coffee beans extract compared to control at 30 min = insulin and glucose 2 h AUC
Johnston et al. 2003 [34]	*n* = 9 (4M/5F); age 26 ± 3.2 y; BMI ≤ 25 kg/m^2^	CO	25 g glucose in 400 mL of (1) caffeinated or (2) decaffeinated coffee	2.5 mmol CGA/L	25 g glucose in 400 mL water	= glucose, insulin at any time point ↑0–30 min IAUC insulin caffeinated coffee vs. DC and control
Jokura et al. 2015 [46]	*n* = 19 M; age 38.1 ± 8.4 y; BMI 21.8 ± 2.2 kg/m^2^	CO	Meal (75 g carb, 28.5 g fat, 8 g protein) with coffee polyphenol extract	355 mg CQAs in 185 mL	Meal (75 g carb, 28.5 g fat, 8 g protein) without coffee polyphenol extract; matched caffeine (54.9 mg)	↓peak postprandial blood glucose at 30 min vs. control ↑ postprandial FMD at 60 min, 120 min, 180 min over 4 h vs. control = postprandial insulin
Kajikawa et al. 2019 [39]	*n* = 18 patients with borderline or stage 1 hypertension (12M/6F); age 56 ± 15 y; BMI 23.2 ± 3.1 kg/m^2^	CO	Single intake of roasted coffee (beverage A)	412 mg CGAs (beverage A)	Single intake of beverage C (no CGAs)	= TG, glucose, insulin, FMD, SBP vs. control ↑TG, glucose, insulin at 1 h and 2 h within beverage A and within control ↑FMD at 1 h and 2 h within beverage A
Lappi et al. 2013 [40]	*n* = 15 (6M/9F); age 57 y; BMI 26 kg/m^2^; healthy but self-reported mild, moderate or severe gastrointestinal symptoms post-ingestion of cereals, particularly rye	CO	Meal including white wheat breads fortified with bioprocessed rye bran (WW + BRB) or with native rye bran (WW + RB) providing 50 g of available starch (meal also included 40 g cucumber, 20 g margarine, 14/15 participants also consumed black coffee or tea)	- WW + BRB: 134.6 mg total FA, 31.3 mg total sinapic acid and 4.7 mg coumaric acid - WW + RB: 117.0 mg total FA, 28.8 mg total sinapic acid and 5.0 mg total coumaric acid	Control meals including wholegrain rye (R) bread (74.0 mg FA + 17.1 mg SA + 2.9 mg CA) or white wheat (WW) bread (6.6 mg FA + 1.7 mg SA + 0.2 mg CA) providing 50 g of available starch (meal also included 40 g cucumber, 20 g margarine, 14/15 participants also consumed black coffee or tea)	= glucose ↓insulin incremental AUC for R bread (control) compared to others
Mills et al. 2017 [25]—Study 1	*n* = 15 M; age 26.3 ± 1.6 y; BMI 23.5 ± 0.5 kg/m^2^	CO	LPC and HPC coffees	89 mg (LPC) and 310 mg (HPC) CGA	Caffeine control: 110 mg caffeine and 0 mg CGA in hot water	↑FMD at 1 h and 5h for 450 mg and 900 mg CGA vs. control = SBP, DBP
Mills et al. 2017 [43]—Study 2	*n* = 24 M; age 23.8 ± 1.4 y; BMI 23.2 ± 1.4 kg/m^2^	CO	450 mg purified 5-CQA + 1 g maltodextrin; 900 mg purified 5-CQA + 1 g maltodextrin	450 mg and 900 mg CGA	1 g maltodextrin (negative control) and 200 mg (−)-epicatechin + 1 g maltodextrin (positive control)	= FMD ↑FMD at 1 h within 450 mg CGA = SBP, DBP
Moser et al. 2018 [44]	*n* = 12 (6M/6F); range age 19–33 y; BMI 23.2 ± 0.7 kg/m^2^	CO	Potato chip product made from (1) white, (2) purple or (3) red potatoes providing 50 g of available carbohydrates (~93 g potato chips)	Total CGAs ranging from 13.4 ± 0.8 to 362.1 ± 9.2 mg/100 g dw	Plain salted wheat crackers providing 50 g of available carbohydrates	↓mean 2 h glucose IAUC and glucose at 20, 40 and 60 min time points to each potato chip vs. control
Mubarak et al. 2012 [32]	*n* = 23 (4M/19F); age 52.3 ± 10.6 y; BMI 25.6 ± 4.7 kg/m^2^	CO	400 mg CGAs in 200 mL of low nitrate water	400 mg CGAs (3-CQA)	Water	↓SBP, DBP (mean of 60, 90, 120 and 150 min) = FMD at 120 min
Ochiai et al. 2014 [31]	*n* = 14 M; age 36.2 ± 7.8 y; BMI 22.7 ± 1.8 kg/m^2^	CO	225 mL of a 75 g glucose-equivalent test solution with coffee polyphenols	600 mg CQAs	225 mL of a 75 g glucose-equivalent test solution	= glucose, insulin, SBP
Ochiai et al. 2015 [30]	*n* = 13 M; age 44.9 ± 1.4 y; BMI 21.9 ± 0.6 kg/m^2^	CO	Coffee bean polyphenols beverage in 100 mL of water followed by a test meal (14 g of protein, 30 g of fat and 58 g of carbohydrates)	600 mg CGAs	Placebo beverage with same taste followed by a test meal (14 g of protein, 30 g of fat and 58 g of carbohydrates)	↓DBP at 1 h vs. placebo ↑FMD at 6 h vs. placebo = glucose, insulin, TG, TC
Rakvaag et al. 2016 [42]	*n* = 11 (4M/7F); age 26.2 ± 0.6 y; BMI 23.2 ± 0.4 kg/m^2^	CO	300 mL of either light (LIR) or dark (DAR) roasted coffee 30 min before 75 g OGTT	- LIR: 399.6 ± 12.5 mg CQA/serving - DAR: 66.6 ± 1.2 CQA mg/serving	300 mL of tap water 30 min before 75 g OGTT	↑insulin 2 h IAUC DAR vs. control = glucose ↑glucose 60 min time point DAR vs. control ↓ISI (Matsuda) DAR and LIR vs. control
Robertson et al. 2015 [43]	*n* = 10 overweight M; age 30.4 ± 14.2 y; BMI 27.8 ± 2.2 kg/m^2^	CO	(i) 2, 4 or 8 g instant DC in 400 mL water with caffeine added in proportion (total 100, 200 or 400 mg caffeine) all with 50 g glucose (ii) 2, 4 and 8 g DC with a standard 100 mg caffeine added to each	47, 94 and 188 mg CQAs/drink	(i) 400 mL water with 50 g glucose (ii) 400 mL water with a standard 100 mg caffeine	(i) ↑2 h IAUC glucose for 2 and 4 g DC vs. control (i) = 2 h IAUC insulin, ISI (Matsuda) (ii) = 2 h IAUC glucose, 2 h IAUC insulin, ISI (Matsuda)
Rosen et al. 2011 [29]	*n* = 14 (7M/7F); age 23.6 ± 0.5 y; BMI 22.0 ± 0.5 kg/m^2^	CO	A portion of one of five whole grain rye test breads (differing in rye varieties) providing 50 g of available starch	FA ranging from 2.2 to 2.6 mg/serving; sinapic acid ranging from 724 to 993 µg/serving	122.7 g of WW bread providing 50 g of available starch	↓3 h IAUC insulin for two rye test breads (Amilo and Rekrut) vs. WW = 3 h IAUC glucose
Schubert et al. 2014 [45]	*n* = 12 (3M/9F); age 26.3 ± 6.3 y; BMI 22.7 ± 2.2 kg/m^2^ (*n* = 10 in glucose analysis)	CO	A standardized breakfast (pancakes, butter, and jam: 48 g carbohydrate, 17 g fat, 15 g protein) at 0 min plus: (i) 225 mL of DC + placebo capsules at 0 and 120 min (ii) 225 mL DC + caffeine capsules at 0 and 120 min	248.9 ± 11.3 mg CGAs per serving	A standardized breakfast (pancakes, butter and jam: 48 g carbohydrate, 17 g fat and 15 g protein) at 0 min plus: (i) Placebo (250 mg Metamucil) capsules and 225 mL water at 0 and 120 min (ii) Caffeine capsules and 225 mL water at 0 and 120 min	= 4.5 h IAUC glucose
Thom 2007 [36]	*n* = 12 (6M/6F); age 24.2 ± 3.2 y; BMI < 25.0 kg/m^2^	CO	(i) 25 g of sucrose and 10 g of coffee made with GCE (ii) 25 g of sucrose and 10 g of normal instant coffee; (iii) 25 g of sucrose and 10 g of DC all in 400 mL water	(i) ~432 mg CGA (ii + iii) ~220 mg CGA	25 g of sucrose in 400 mL of water	↓2 h IAUC glucose AUC for GCE vs. control only
Van Dijk et al. 2009 [38]	*n* = 15 overweight M; age 39.9 ± 16.5 y; BMI 27.6 ± 2.2 kg/m^2^	CO	Either (i) 12 g DC (ii) 1 g CGA 30 min before a 75 g oral glucose	(i) 264 mg CGA (ii) 1 g CGA	1 g mannitol 30 min before a 75 g oral glucose	= 2 h IAUC glucose and insulin ↓glucose and insulin at 15 min CGA vs. control
Ward et al. 2016 [33]	*n* = 16 (6M/10F); age 58.9 ± 8.2 y; BMI 24.7 ± 3.3 kg/m^2^	CO	200 mL warm water with either 450 mg and 900 mg purified 5-CGA 2 h after a standardized breakfast (2 pieces of white bread with cheese, tub of plain yoghurt and water)	450 and 900 mg CGA	200 mL warm water with either 0 mg purified 5-CGA (control group) or 200 mg purified (−)-epicatechin (positive control) 2 h after a standardized breakfast (2 pieces of white bread with cheese, tub of plain yoghurt and water)	↑continuous FMD vs. control = BP, peak FMD

Legend: AUC: Area under the curve; BMI: Body mass index; BP: Blood pressure; BRB: Bioprocessed rye bran; bw: Body weight; CGAs: Chlorogenic acids; CO: Cross-over design; DAR: Dark roasted coffee; DBP: Diastolic blood pressure; DC: Decaffeinated coffee; dw: Dry weight; EDGCB: Decaffeinated green coffee beans extract; F: Females; FA: Ferulic acid; FMD: Flow mediated dilation; GCE: Green coffee; HPC: High polyphenol coffee; IAUC: Incremental area under the curve; ISI: Insulin sensitivity index; LIR: Light roasted coffee; LPC: Low polyphenol coffee; M: Males; OGTT: Oral glucose tolerance test; SBP: Systolic blood pressure; WW: Whole wheat; =: No statistically significant effect; ↑: Significant increase; ↓: Significant decrease. Age and BMI are mean ± SD.

**Table 2:** Characteristics of the considered chronic intervention studies investigating the effect of HCA-rich foods on cardiometabolic markers.

Reference	Subjects	Study Design	Intervention	Dose of HCAs	Control/Placebo	Main Findings
Agudelo-Ochoa et al. 2016 [63]	*n* = 75 (38M/37F); age 38.5 ± 9 y; BMI 24.1 ± 2.6 kg/m^2^; overall healthy	P	400 mL coffee/d containing a medium (MCCGA) or high (HCCGA) CGA content for 8 weeks	MCCGA = 420 mg/400 mL; HCCGA = 780 mg/400 mL (each with ~193 mg/d caffeine)	No consumption of coffee or placebo	=TC, LDL, HDL, TG, FMD, BP
Bumrungpert et al. 2018 [59]	*n* = 48 with hyperlipidemia (6M/42 F); age 48.7 ± 7.6 y (FA) and 45.9 ± 7.8 y (placebo); BMI 25.9 ± 5.3 kg/m^2^ (FA) and 26.6 ± 6.2 kg/m^2^ (placebo)	P	2 capsules of FA/day for 6 weeks	1000 mg FA/day	2 placebo capsules/day for 6 weeks	↓TC, LDL, and TG ↑HDL = SBP, DBP, glucose, BMI, WC compared to control
Haidari et al. 2017 [61]	64 obese F; age 36.1 (GCE) and 35.7 y (placebo); BMI 26.4 ± 2.5 kg/m^2^ (GCE) and 26.4 ± 2.5 kg/m^2^ (placebo)	P	One capsule of 400 mg GCE/day for 8 weeks	180 mg CGA/capsule	One placebo capsule of 400 mg starch/day for 8 weeks	↓BMI, TC, LDL = HDL, TG, glucose, insulin in the GCE group + compared to control
Kempf et al. 2015 [57]	*n* = 116 overweight (39M/77F); age 50.6 ± 11.8 y (M-coffee) and 48.0 ± 12.7 y (D-coffee); BMI 32.4 ± 4.4 kg/m^2^ (M-coffee) and 33.9 ± 4.6 kg/m^2^ (D-coffee)	CO	4–5 servings/day of 7.5 g MRC in 125 mL water for 3 months	19.31 ± 0.28 mg/L CGA	7.5 mg/L dark roast coffee (10.01 ± 0.28 mg/L CGA; 1.20 ± 0.03 mg/L *N*-methylpyridinium; caffeine-matched) in 125 mL water for 3 months	↓SBP in control vs. M-coffee ↑TG within control ↑HDL within M-coffee = BMI, DBP, glucose, insulin, HOMA-IR, TC, LDL, HbA1c between M-coffee and control
Kozuma et al. 2005 [50]	*n* = 117 M with mild hypertension -Group 46 mg (*n* = 29): age 42.9 ± 8.2 y; BMI 25.2 ± 4.0 kg/m^2^ -Group 93 mg (*n* = 28): age 43.3 ± 8.3 y; BMI 24.4 ± 2.6 kg/m^2^ -Group 185 mg (*n* = 31): age 43.4 ± 8.4 y; BMI 25.1 ± 3.6 kg/m^2^ -Placebo (*n* = 29): age 43.1 ± 9.1 y; BMI 24.0 ± 3.1 kg/m^2^	P	Soy sauce-flavored instant cup soup containing 46 mg, 93 mg, or 185 mg of GCE, once a day for 28 days	25 mg, 50 mg, and 100 mg CGAs, respectively	Soy sauce-flavored instant cup soup without GCE, once a day for 28 days	↓SBP and DBP in all group compared to baseline, significant for 93 and 185 g groups compared to placebo ↓TC, LDL within 185 mg group = BMI, TG, HDL
Lecoultre et al. 2014 [52]	*n* = 10 M; age 23 ± 2 y; BMI 22.6 ± 0.6 kg/m^2^; healthy	CO	4 cups of (i) caffeinated coffee high in CGA (C-HCA), (ii) DC high in CGA (D-HCA), or (iii) DC regular in CGA (D-RCA) daily for 14 days, with 4 g fructose/kg day the last 6 days	-C-HCA: 9% CGAs -D-HCA: 9% CGAs -D-RCA: 3% CGAs	No coffee + 4 g fructose/kg d (high fructose only) in the last 6 days	= TG, glucose, insulin ↑TG after control
Martínez-López et al. 2018 [28] *	*n* = 52: *n* = 25 NC (10M/15F) and *n* = 27 HC (10M/17F); age 26.6 ± 7.7 y (NC-F), 24.7 ± 5.8 y (NC-M), 33.3 ± 10.2 y (HC-F), 34.8 ± 9.2 y (HC-M); BMI 21.9 ± 2.5 kg/m^2^ (NC-F), 24.2 ± 2.8 kg/m^2^ (NC-M), 21.4 ± 2.5 kg/m^2^ (HC-F), 24.9 ± 2.3 kg/m^2^ (HC-M)	CO	6 g/day of soluble green/roasted (35:65) coffee for 8 weeks	510.6 mg/day HCAs (~344 mg/d CGA) + ~120 mg caffeine	Control beverage (water or an isotonic drink) for 8 weeks	↓TC, LDL, TG =HDL ↓TC, LDL, TG in HC only = TC, LDL, TG in NC only
Ochiai et al. 2004 [55]	*n* = 20 M; age 37.2 ± 1.6 y (test) and 34.8 ± 2.3y (placebo); BMI 24.7 ± 1.6 kg/m^2^ (test) and 23.8 ± 0.6 kg/m^2^ (placebo); healthy	P	1 bottle of 125 mL test drink containing GCE for 4 months	140 mg CGA/serving (28%)	125 mL GCE-free drink	= SBP, DBP, TC, LDL, HDL, TG, glucose, insulin
Ochiai et al. 2009 [64]	*n* = 21 with mild hypertension and vascular failure; range age 30–64 y; BMI 24.2 ± 0.9 kg/m^2^ (active group) and 24.2 ± 1.1 kg/m^2^ (placebo group)	P	1 can (184 mL) of active beverage (canned coffee) for 8 weeks	Active beverage: 300 mg CGA/serving (reduced in HHQ)	1 can (184 mL) of placebo beverage for 8 weeks (reduced in HHQ and CGA; caffeine-matched)	↓SBP = DBP, BMI, glucose, HbA1c, TG, LDL, HDL = DBP, glucose, BMI, FMD, TC, LDL, HDL, TG
Rezazadeh et al. 2018 [58]	68 patients with MetS (19M/49F); age 38.9 ± 6.9 y; BMI 34.3 ± 4.3 kg/m^2^	P	1800 mg/day ALE as four tablets for 12 weeks	4%–5% CGAs	4 placebo tablets/day	↓TG, TC, LDL within ALE =HDL ↓TG in ALE vs. control
Riedel et al. 2014 [56]	*n* = 84 (46M/38F); age 25.6 ± 5.8 y; BMI 22.9 ± 1.9 kg/m^2^; healthy	CO	750 mL (6 coffee pads at 7.5 g) of MRC per day for 4 weeks	19.2 ± 0.3 mg/g CGA	750 mL of DRC (10.0 ± 0.3 mg/L CGA; 1.20 ± 0.0 mg/L *N*-methylpyridinium; caffeine-matched) per day for 4 weeks	↑TC within MRC only ↑insulin, HOMA-IR within MRC and vs. DRC (control)↑HDL within MRC and DRC, but greater in DRC (control) =LDL, TG and glucose
Robertson et al. 2018 [72]	*n* = 27: *n* = 19 (10M/9F) in the coffee group; range age 18–42; BMI 23.4 ± 3.0 kg/m^2^; *n* = 8 (4M/4F) in the control group; range age 19–41; BMI 22.9 ± 3.1 kg/m^2^; overall healthy	P	Four cups/day instant coffee for 12 weeks	43.6 mg CGAs/cup	No coffee/caffeine for 12 weeks	= fasting glucose, insulin, TG, BMI
Rondanelli et al. 2013 [54]	*n* = 92 with mild hypercholesterolemia (41M/51F); age 54.2 ± 6.6 y (intervention) and 53.8 ± 9.0 y (placebo); BMI 25.8 ± 3.9 kg/m^2^ (intervention) and 24.8 ± 1.6 kg/m^2^ (placebo)	P	Tablets of 250 mg ALE twice a day for 8 weeks	>20% CQAs	Placebo tablets of 250 mg twice a day for 8 weeks	↑HDL within and between groups ↓LDL and TC within and between groups = TG, glucose
Rondanelli et al. 2014 [62]	*n* = 55 overweight with IFG (25M/30F) age 54.1 ± 9.8 y; BMI 30.7 ± 3.8 kg/m^2^	P	600 mg/day extract of Cs (30%–60% CQAs) tablets for 8 weeks	CQAs between 30% and 60%	Placebo tablets for 8 weeks	↓BMI, glucose, HOMA, TC, and LDL within Cs and vs. control ↓HbA1c within Cs only = insulin, HDL, TG, WC
Roshan et al. 2018 [67]	*n* = 43 with MetS (10M/33F); age 52.8 ± 9.8 y (CGE) and 52.0 ± 8.7 y (placebo); BMI 31.6 ± 3.6 kg/m^2^ (CGE) and 31.2 ± 4.9 kg/m^2^ (placebo)	P	Two decaffeinated CGE capsules of 400 mg/day for 8 weeks	372 mg CGA/day (186 mg/capsule)	Two placebo capsules (starch)/day for 8 weeks	↓SBP, fasting glucose, HOMA-IR, and WC compared to placebo = DBP, insulin, HbA1c, BMI, TC, LDL, HDL, TG
Sarriá et al. 2016 [27] *	*n* = 52 (20M/32F); age 29.8 ± 8.9 y (M) and 29.4 ± 9.5 y (F); BMI 24.8 ± 2.7 kg/m^2^ (M) and 21.7±2.5 kg/m^2^ (F)	CO	6 g/day of soluble green/roasted (35:65) coffee for 8 weeks	510.6 mg/day HCAs (~344 mg/day CGA) + ~120 mg caffeine	Water or an isotonic drink	↓glucose and HOMA-IR ↑QUICKI
Sarriá et al. 2018 [26] *	*n* = 52: *n* = 25 NC (10M/15F) and *n* = 27 HC (10M/17F); age 26.6 ± 7.7 y (NC-F), 24.7 ± 5.8 y (NC-M), 33.3 ± 10.2 y (HC-F), 34.8 ± 9.2 y (HC-M); BMI 21.9 ± 2.5 kg/m^2^ (NC-F), 24.2 ± 2.8 kg/m^2^ (NC-M), 21.4 ± 2.5 kg/m^2^ (HC-F), 24.9 ± 2.3 kg/m^2^ (HC-M)	CO	6 g/day of soluble green/roasted (35:65) coffee for 8 weeks	510.6 mg/day HCAs (~344 mg/day CGA) + ~120 mg caffeine	Water or an isotonic drink	↓SBP, DBP ↓glucose, HOMA-IR and TG = HDL, insulin, waist circumference ↓SBP, DBP, HOMA-IR, TG in HC only = HDL, glucose, insulin in HC only ↓HOMA-IR in NC only = SBP, DBP, TG, HDL, glucose, insulin in NC only
Shahmohammadi et al. 2017 [60]	*n* = 44 (22M/22F) patients with NAFLD; age 41.4 ± 7.7 y (GCE) and 44.5 ± 5.2 y (placebo); BMI 31.3 ± 2.6 kg/m^2^ (GCE) and 31.5 ± 2.2 kg/m^2^ (placebo)	P	1 g GCE/day for 8 weeks + advice on 30 min/day exercise	500 mg CGAs/day	1 g placebo/day for 8 weeks + advice on 30 min/day exercise	↓TC, TG, fasting blood glucose, HOMA-IR = HDL, LDL, insulin, WC, BMI for GCE vs. control
Soga et al. 2013 [69]	*n* = 18 M; age 36.1 ± 7:4 y; BMI 21.9 ± 0.6 kg/m^2^ (CGA) and 22.0 ± 0.5 kg/m^2^ (control); healthy	CO	185 mL of a test beverage with CGAs daily for 4 weeks	329 mg CGAs/serving	185 mL of test beverage without CGAs for 4 weeks; caffeine-matched	↓glucose within CGA = BMI
Vinson et al. 2012 [73]	*n* = 18 hypertensive (7M/11F); age 54 ± 10 y; BMI 29.4 ± 6.4 kg/m^2^	CO	6–8 purple potatoes for 4 weeks	2.72 mg/g dw CGA; 0.147 mg/g dw neoCGA	No potatoes for 4 weeks	↓DBP within purple potatoes = SBP, glucose, HbA1c, TC, HDL, TG
Vitaglione et al. 2015 [71]	*n* = 68 overweight/obese otherwise healthy (23M/45F); age 40 ± 2 y (WG) and 37 ± 2 y (control); BMI 30.0 ± 0.5 kg/m^2^ (WG) and 29.5 ± 0.4 kg/m^2^ (control)	P	70 g/d (3 biscuits/d) of WG products for 8 weeks	96.7 mg FA/serving; 26.5 mg SA/serving; 9.4 mg CA/serving	1 package (33 g) of crackers and 3 slices of toasted bread for 8 weeks	= BMI, WC, glucose, TC, HDL, TG
Watanabe et al. 2006 [66]	*n* = 28 with mild hypertension (11M/17F); age 52 ± 11 y (CGA) and 51 ± 8 y (placebo); BMI 23.8 ± 3.3 kg/m^2^ (CGA) and 25.0 ± 3.5 kg/m^2^ (placebo)	P	125 mL/day fruit and vegetable juice mixed with GCE for 12 weeks	140 mg CGA/day	125 mL/day fruit and vegetable juice for 12 weeks	↓SBP and DBP within GCE ↓SBP GCE vs. control = BMI, TC, LDL, HDL, TG, glucose
Wedick et al. 2011 [65]	*n* = 45 overweight (16M/29F); age 40 y; BMI 29.5 kg/m^2^	P	5 cups (177 mL each) per day of (1) DC or (2) caffeinated coffee for 8 weeks	(1) 216 mg/5 cups of DC (2) 302 mg/5 cups for caffeinated coffee	No coffee (i.e., water) for 8 weeks	= BMI, WC, SBP, LDL, HDL, TG, glucose, insulin and HOMA-IR ↓DBP within DC
Yamaguchi et al. 2008 [53]	*n* = 183 with mild hypertension (91M/92F); age 49.4 ± 10.1 y; BMI 25.4 ± 3.8 kg/m^2^	P	One can (184 mL)/day of zero-dose, low-dose, middle-dose, high-dose coffee for 4 weeks	82 mg (low-dose), 172 mg (middle-dose) and 299 mg (high-dose) CGA/can	0 mg (zero-dose) coffee (0 mg CGA)	↓SBP and DBP in low, middle and high-dose = LDL, TG ↑HDL and TC within middle-dose ↓BW within middle and high dose
Zuñiga et al. 2017 [51]	*n* = 30 (5M/25F) patients with IGT; age 43 ± 11 y (CGA) and 45 ± 9 y (placebo); BMI 32.6 ± 2.4 kg/m^2^ (CGA) and 32.1 ± 2.5 kg/m^2^ (placebo)	P	Oral CGAs from GCE, three times per day for 12 weeks	1200 mg CGAs/day	Oral placebo, three times per day for 12 weeks	↓BMI, WC, TG, TC, LDL, glucose = SBP, DBP, HDL, HbA1c within CGA

Legend: ALE: Artichoke leaf extract; AUC: Area under the curve; BMI: Body mass index; BP: Blood pressure; CA: Coumaric acid; CGA: Chlorogenic acid; CO: Cross-over design; Cs: *Cynara scolymus* (artichoke); DC: Decaffeinated coffee; D-coffee: Dark roast coffee; DBP: Diastolic blood pressure; DRC: Dark roast blend coffee; dw: Dry weight; F: Females; FA: Ferulic acid; FMD: Flow mediated dilation; GCE: Green coffee extract; HbA1c: Glycated hemoglobin; HC: Hypercholesterolemic; HCAs: Hydroxycinnamic acids; HDL: High density lipoprotein; HHQ: Hydroxyhydroquinone; HOMA: Homeostatic model assessment; IFG: Impaired fasting glucose; IGT: Impaired glucose tolerance; IR: Insulin resistance; LDL: Low density lipoprotein; M: Males; M-coffee: Medium roast coffee; MetS: Metabolic syndrome; MRC: Medium roast blend coffee; NAFLD: Non-alcoholic fatty liver disease; NC: Normocholesterolemic; P: Parallel design; RCT: Randomized controlled trial; SA: Sinapic acid; SBP: Systolic blood pressure; TC: Total cholesterol; TG: Triglycerides; WC: Waist circumference; WG: Whole grain; =: No statistically significant effect; ↑: Significant increase and ↓: Significant decrease. Age and BMI are mean ± SD. * These publications correspond to the same RCT.

**Table 3:** Main findings of studies investigating the effect of HCA-rich foods on cardiometabolic markers and considering determinants of inter-individual variability.

Reference	Determinant of Inter-Individual Variability	Main Findings
**Acute studies**		
Ioakeimidis et al. 2018 [49]	Dietary pattern (habitual and non habitul coffee consumers)	= BP between habitual and non-habitual consumers
Iwai et al. 2013 [35]	Pathophysiological status (high glycemic response vs. total mean glycemic response)	↓glucose after both 100 mg 300 mg EDGCB in high glycemic response group
Jokura et al. 2015 [46]	Pathophysiological status (lower or higher insulinogenic index)	↓blood glucose after CPE compared to the placebo in study participants with a lower insulinogenic index ↑postprandial FMD after CPE compared to the placebo in study participants with either lower or higher insulinogenic index
**Chronic studies**		
Martínez-López et al. 2018 [28] *	Pathophysiological status (normocholesterolemia vs. hypercholesterolemia)	↓TC, LDL, TG only in HC
Sarriá et al. 2018 [26] *	Pathophysiological status (normocholesterolemia vs. hypercholesterolemia)	↓SBP, DBP only in HC ↓HOMA-IR in both HC and NC separately = glucose in either HC or NC separately
Rezazadeh et al. 2018 [58]	-Taq IB polymorphism in cholesteryl ester transfer protein gene (B1B1 vs. B2 allel) -Sex (men vs. women with the selected polymorphisms)	Similar changes in lipid profile among Taq IB genotype compared to placebo ↓LDL in men with B1B1 genotype after ALE
Rondanelli et al. 2013 [54]	Sex (men vs. women) Stratified by median HDL	↑HDL in men only ↓LDL, TC in men and women ↓ TG in women only ↑ HDL in men in both strata ↑HDL in women in ≤median HDL strata

Legend: ALE: Artichoke leaf extract; CPE: Coffee polyphenol extract; DBP: Diastolic blood pressure; EDGCB: Decaffeinated green coffee beans extract; FMC: Flow mediated dilation; HC: Hypercholesterolemic; HDL: High density lipoprotein; HOMA: Homeostatic model assessment; IFG: Impaired fasting glucose; IGT: Impaired glucose tolerance; IR: Insulin resistance; LDL: Low density lipoprotein; NC: Normocholesterolemic; PP: Pulse pressure; TC: Total cholesterol; SBP: Systolic blood pressure; TG: Triglycerides; =: No statistically significant effect; ↑: Significant increase; ↓: Significant decrease. * These publications correspond to the same RCT.

## Supplementary Materials

The following are available online at https://www.mdpi.com/2072-6643/11/8/1805/s1: Supplementary Figure S1: Risk of bias graph: review authors’ judgments about each risk of bias item presented as percentages across all included acute (A) and chronic (B) studies, Supplementary Figure S2: Risk of bias summary: review authors’ judgments about each risk of bias item for each included acute (A) and chronic (B) study. Supplementary Table S1: PRISMA checklist. Supplementary Table S2: Literature search strategy used for each considered database. Supplementary Table S3: PICOS criteria for the inclusion of the intervention studies.
